# Impact of early-onset preeclampsia on feeding tolerance and growth of very low birth weight infants during hospitalization

**DOI:** 10.1590/1984-0462/2023/41/2021203

**Published:** 2022-09-09

**Authors:** Simone Manso de Carvalho Pelícia, Saskia Maria Wiegerinck Fekete, Jose Eduardo Corrente, Ligia Maria Suppo de Souza Rugolo

**Affiliations:** a Universidade Estadual Paulista, Faculdade de Medicina de Botucatu – Botucatu, SP, Brazil.

**Keywords:** Preeclampsia, Infant, premature, Gastrointestinal tract, Enteral feeding, Pré-eclâmpsia, Recém-nascido prematuro, Trato gastrointestinal, Nutrição enteral

## Abstract

**Objective:**

The provision of adequate enteral nutrition to preterm infants is a great challenge, and preeclampsia (PE) may have a detrimental effect on the safety of nutrition supply. This study aims to investigate the influence of early-onset PE on preterm infants’ enteral feeding tolerance and growth during hospitalization.

**Methods:**

This is a prospective study with 55 preterm infants <34 weeks born to PE mothers matched by gestational age with 55 preterm infants born to normotensive mothers from 2013 to 2016. We evaluated maternal, gestational, and neonatal clinical data. The outcomes were feeding intolerance and growth during hospitalization. Comparison between groups was performed by Student’s *t*-test or Mann-Whitney U test, chi-square test, or Fisher’s exact test. Multiple logistic regression was used to investigate whether PE was an independent risk factor for feeding intolerance.

**Results:**

The mean gestational age was 30 weeks. Preterm infants of mothers with PE had lower birth weight and were smaller at discharge. Feeding intolerance was frequent, but necrotizing enterocolitis was rare in this sample (PE=4% vs. control=2%) with no difference between groups. Preterm infants of mothers with PE had worse growth outcomes; however, PE was not an independent risk factor for feeding intolerance. The increase in gestational age was a protective factor, and being born small for gestational age (SGA) increased the risk of feeding intolerance by six times.

**Conclusions:**

Preterm infants of mothers with early-onset PE were more likely to be born SGA and had a worse growth trajectory during hospitalization. In adjusted analyses, however, low gestational age and SGA were independent predictors of feeding intolerance.

## RESUMO

**Objetivo:**

A nutrição enteral adequada para recém-nascidos prematuros é um grande desafio, e a pré-eclâmpsia (PE) pode comprometer a segurança da oferta alimentar. O objetivo deste estudo é investigar a influência da PE de início precoce na tolerância alimentar e no crescimento de prematuros durante a hospitalização.

**Métodos:**

Estudo prospectivo, com 55 prematuros <34 semanas de mães com PE pareados por idade gestacional e com 55 prematuros de mães normotensas, de 2013 a 2016. Foram avaliados dados clínicos maternos, gestacionais e neonatais. Os desfechos foram intolerância alimentar e crescimento durante a hospitalização. Na comparação entre grupos, utilizaram-se teste *t* de Student ou de Mann-Whitney e teste qui-quadrado ou exato de Fisher. Regressão logística múltipla foi usada para investigar se a PE é fator de risco para intolerância alimentar.

**Resultados:**

A idade gestacional média foi de 30 semanas. Prematuros de mães com PE tiveram menor peso ao nascer e eram menores na alta. A intolerância alimentar foi frequente, mas a enterocolite necrosante foi rara nesta amostra (PE=4% vs. controle=2%), sem diferença entre grupos. Prematuros de mães com PE tiveram pior crescimento, mas a PE não foi fator independente de risco para intolerância alimentar. O aumento da idade gestacional foi fator de proteção, e nascer pequeno para a idade gestacional (PIG) aumentou em seis vezes o risco de intolerância alimentar.

**Conclusões:**

Prematuros de mães com PE de início precoce tiveram maior probabilidade de nascer PIG e pior trajetória de crescimento na hospitalização. Em análises ajustadas, baixa idade gestacional e PIG foram preditores independentes de intolerância alimentar.

## INTRODUCTION

Preeclampsia (PE) is the main medical indication for premature birth.^1^ This disease is associated with important vascular changes and compromised placental and fetal blood flow, but its repercussions on the newborn are not yet well established.^2^ There are studies showing a higher risk of perinatal morbidity and mortality,^3^ while others have not reported differences in short-term prognosis.^4^ It is also unclear whether the neonatal outcome is directly associated with the maternal disease or related to the effects of its treatment with MgSO_4_ on intestinal blood flow.^5^ One aspect of concern is the association of PE with necrotizing enterocolitis (NEC), which could be explained by impairment in uteroplacental blood flow leading to intestinal ischemia and fetal pro-inflammatory state.^2–4,6^

Preterm birth results in the early transition from transplacental nutrition to enteral feeding through an immature gastrointestinal tract and is itself a risk factor for feeding intolerance and NEC.^7^ Thus, the provision of adequate and safe enteral nutrition to preterm infants is a major challenge in clinical practice.

The goal in the nutritional care of preterm infants is to provide adequate postnatal growth and neurodevelopmental outcome. However, there are several difficulties that limit nutritional supply, especially feeding intolerance, which raises concern about the risk of NEC. So, a slower rate of feed advancement could be a safe alternative, but it prolongs the need for parenteral nutrition and its complications and does not decrease the risk of NEC.^8,9^

Intrauterine and postnatal circulatory disorders are described in infants of PE mothers, including decreased intestinal blood flow;^10^ however, the clinical consequences of these abnormalities are still poorly studied.^10,11^ We hypothesized that PE negatively affects the preterm infants’ enteral feeding tolerance and growth. This study was designed to address the effect of early-onset PE on the preterm infants’ enteral feeding tolerance and growth during hospitalization.

## METHOD

A prospective observational study with preterm infants <34 weeks, born to mothers with PE, matched by the same gestational week with preterm infants of normotensive mothers (control group sequentially enrolled), born and admitted to the neonatal intensive care unit (NICU) of a tertiary University Hospital, from June 2013 to May 2016.

The study was approved by the research ethics committee and informed consent form was obtained from all participants.

Based on the proportion of feeding problems in preterm infants of PE mothers reported by Ersch et al.,^11^ with a test power of 90% and type I error of 0.05, a minimum number of 40 participants in each group was determined.

Preeclampsia was defined as hypertension accompanied by proteinuria (≥300mg in 24-h urine) after the 20th week of gestation in previously normotensive women. The disease was classified as early-onset PE when diagnosed before 34 weeks’gestation.^12^

Both groups were recruited concurrently, and the inclusion criteria were as follows: single pregnancy and inborn premature infants <34 weeks of gestation without malformations or congenital infections. Deaths in the first week of life were excluded.

The following maternal and gestational data were evaluated: use of antenatal steroids (≥1 dose) and magnesium sulfate (for treatment of maternal disease), premature labor, fetal distress (tachycardia, bradycardia, late or variable decelerations in cardiotocography), premature rupture of membranes, chorioamnionitis (antepartum fever and two or more criteria: uterine tenderness, foul-smelling amniotic fluid, maternal or fetal tachycardia), and type of delivery. The neonatal variables included gestational age (defined by the best obstetric estimate, preferably first-trimester ultrasound or precise date of last menstruation), birth weight, gender, small for gestational age (SGA) (weight <10th percentile in the intrauterine growth curve of Fenton and Kim),^13^ resuscitations at birth (need for a bag and mask ventilation), 5-min Apgar score, Score for Neonatal Acute Physiology with Perinatal Extension (SNAPPE) severity score (≥40 indicative of high severity),^14^ vasoactive drugs used in the first 72h of life (indicative of hemodynamic instability), umbilical artery or vein catheter, use of antibiotics in the first 72h of life, and hemodynamically significant patent ductus arteriosus (PDA) (diagnostic by functional echocardiography in the first 72h of life).

According to our institutional guidelines, stable preterm infants <34 weeks start enteral feeding with 10–20mL/kg/day of mother’s own milk or human donor milk on the first day of life. Feeds are given via gavage with 4–6h intervals and increased by 10–30mL/kg/day according to neonate tolerance. Feed volumes can be altered or stopped at the clinician’s discretion if there are symptoms of intolerance. When an enteral intake of 100mL/kg/day is tolerated, human milk is fortified by adding a commercially available fortifier.

In our service, parenteral nutrition starts on the first day of life with a glucose solution, electrolytes, and amino acids of 3g/kg/day. On the second day, the lipid supply starts with 1–1.5g/kg/day (according to birth weight <750 or ≥750g), followed by a daily increase of 1g/kg up to a maximum of 3g/kg/day of lipids and 4g/kg/day of amino acids. Anthropometric measurements are performed twice a week. Infant weight and length were measured by a trained nurse, using an electronic weight balance and a length board. Head circumference (HC) was obtained with an inelastic measuring tape at the maximal occipital frontal circumference.

The primary outcome measure was feeding intolerance and NEC. Feeding intolerance was defined by one or more of these findings: abdominal distension (↑2cm or more in abdominal circumference), gastric residuals (>20% of the volume administered in ≥2 feedings), vomiting, and more than one feeding interruption episode by day. NEC was considered when the stage is 2 or higher according to Bell’s criteria.^14^ The feeding outcome was evaluated by periods: first 3 days, 4–7 days, and between 8–28 days of life.

The secondary outcome was growth during hospitalization, assessed by weight, length, and HC Z scores at birth and at discharge. The independent nutritional data included day at first feed, type of milk (e.g., breast milk, donor human milk, and preterm formula), days of parenteral nutrition, days to reach full enteral feeding (≥120mL/kg/day), and feeding at hospital discharge (e.g., exclusive breastfeeding, mixed feeding, and formula feeding).

Comparison between groups for numerical variables was performed by Student’s t-test for normal distribution or by Mann-Whitney U test for non-normal distribution. For categorical variables, the chi-square test or Fisher’s exact test was used. To investigate whether PE is an independent risk factor for feeding intolerance, stepwise multiple logistic regression was used. Two models were constructed: one had as outcome symptoms of feeding intolerance and the other has feeding interruption. The analyses were performed using the statistical software SAS for Windows (version 9.4), and the significance level was considered 5%.

## RESULTS

A total of 280 very low birth weight (VLBW) infants were born in our service during the 3 years period of recruitment, and 261 of them were <34 weeks’ gestation. Of these eligible patients, 179 met the inclusion criteria, and 70 were born of PE mothers (12 with congenital anomalies, 11 with congenital infections, and 59 multiple gestations were not included). A total of 55 premature infants born of mothers with PE could be matched by the same week of gestational age with 55 premature infants born of normotensive mothers (control group) and were studied. There was no death in the first week of life.

In the PE group, eight mothers had PE superimposed on chronic hypertension; however, this maternal condition was not associated with the obstetric diagnosis of fetal growth restriction, and only one neonate in this subgroup was SGA.

Table 1 shows the main maternal and gestational characteristics.

**Table 1:** Gestational and birth data in the preeclampsia and control groups.

	PE n=55	Control n=55	p-value
Antenatal steroids, n (%)	52 (94.5)	42 (76)	0.015
MgSO_4_, n (%)	39 (71)	12 (22)	<0.001
Premature labor, n (%)	10 (18)	42 (76)	<0.001
PROM>18h, n (%)	2 (4)	12 (22)	0.010
Chorioamnionitis, n (%)	1 (2)	8 (14.5)	0.037
Fetal distress, n (%)	16 (29)	12 (22)	0.511
Cesarean section, n (%)	52 (94.5)	24 (44)	<0.001
Gestational age (x±SD) (Minimum–Maximum)	30±2 (27–33)	30±2 (27–33)	1.000
Birth weight (x±SD) (Minimum–Maximum)	1240±345 (680–2175)	1525±370 (865–2210)	<0.001
Resuscitation at birth, n (%)	32 (58)	20 (36)	0.036
5-min Apgar <7, n (%)	7 (13)	7 (13)	0.775
Female, n (%)	37 (67)	25 (45)	0.034
SGA, n (%)	12 (22)	3 (5.5)	0.026
SNAPPE, median (IQ)	11 (0–25)	8 (0–18)	0.282
SNAPPE ≥40, n (%)	8 (14.5)	2 (4)	0.097

PE: preeclampsia, PROM: premature rupture of membrane, SGA: small for gestational age, SNAPPE: Score for Neonatal Acute Physiology with Perinatal Extension, IQ: interquartile. x±SD: mean ± standard deviation, x±SD denotes mean ± standard deviation.

Neonatal morbidity was low and did not differ between the groups. Only one-third of the premature infants had umbilical catheters, 10% needed vasoactive drugs, 30% used antibiotics in the first 72h, and 18% had hemodynamically significant PDA.

Table 2 shows the nutritional data in the two groups.

**Table 2:** Nutritional data of preterm infants of mothers with preeclampsia and control.

	PE (n=55)	Control (n=55)	p-value
Day at first feed (x±SD)	2.4±2.4	2.0±2.2	0.345
Mother’s own breast milk at first feed (%)	70	71	1.000
Mother’s milk at feeding advancement (%)	69	70	0.886
Day of life on full enteral feeding (x±SD)	13±8	9±7	0.029
Parenteral nutrition days (x±SD)	13±10	9±7	0.028
Exclusive breastfeeding at discharge (%)	52	53	0.912
Breastfeeding+formula at discharge (%)	29	25	0.860

x±SD: mean ± standard deviation, x±SD denotes mean ± standard deviation, PE: preeclampsia.

Feeding intolerance was frequent in both groups, with no differences between them. However, NEC was uncommon in this sample, as shown in Table 3.

**Table 3:** Feeding intolerance in preterm infants of mothers with preeclampsia and controls.

Signals of feeding intolerance		PE (n=55)	Control (n=55)	p-value
Days 1–3				
	None (%)	22 (40)	22 (40)	1.000
	Gastric residuals (%)	29 (53)	33 (60)	0.564
	Abdominal distension or vomiting (%)	3 (5.5)	0 (-)	0.242
	Feeding interruption (%)	13 (24)	7 (13)	0.216
Days 4–7				
	None (%)	24 (44)	29 (53)	0.445
	Gastric residuals (%)	22 (40)	25 (45)	0.700
	Abdominal distension or vomiting (%)	5 (9)	0 (0)	0.067
	Feeding interruption (%)	13 (24)	6 (10)	0.130
	Days 8–28	n=54 *	n=55	
	None (%)	33 (61)	31 (56)	0.757
	Gastric residuals (%)	17 (31)	22 (40)	0.467
	Abdominal distension or vomiting (%)	4 (7)	3 (5)	0.980
	Feeding interruption (%)	8 (15)	7 (13)	0.969
	Necrotizing enterocolitis (%)	2 (4)	1 (2)	0.987

* Death of an 18-day-old (grade 4 intraventricular hemorrhage with hydrocephalus). PE: preeclampsia.

Potential factors associated with feeding intolerance were investigated by multiple logistic regression models, including perinatal variables that differed between groups in univariate analysis, as well as those of clinical relevance and interest in this study: PE, gestational age, severity score (SNAPPE>40), umbilical catheter, and hemodynamically significant PDA.

The multivariate analysis showed that PE was not an independent risk factor for feeding intolerance. The risk factors for feeding intolerance and feeding interruption episodes in the first 28 days of life are illustrated in Table 4.

**Table 4:** Independent risk factors for feeding intolerance and interruption.

Period	Outcome	Variable	OR (95%CI)	p-value
Days 1–3	Feeding intolerance	Gestational age	0.80 (0.65–0.98)	0.036
	Feeding interruption	Gestational age	0.63 (0.42–0.92)	0.019
		SNAPPE >40	1.04 (1.00–1.07)	0.050
Days 4–7	Feeding intolerance	Gestational age	0.75 (0.60–0.93)	0.009
	Feeding interruption	Gestational age	0.53 (0.33–0.85)	0.009
		Maternal MgSO_4_	0.20 (0.05–0.92)	0.039
Days 8–28	Feeding intolerance	Gestational age	0.59 (0.46–0.77)	<0.001
		SGA	5.99 (1.64–21.82)	0.008

SGA: small for gestational age; OR: *Odds Ratio*; 95%CI: 95% confidence interval.

There was no difference between PE versus control groups regarding the length of stay (39±19 vs. 33±19 days; p=0.122) and anthropometric measurements at discharge.

**Figure 1:**
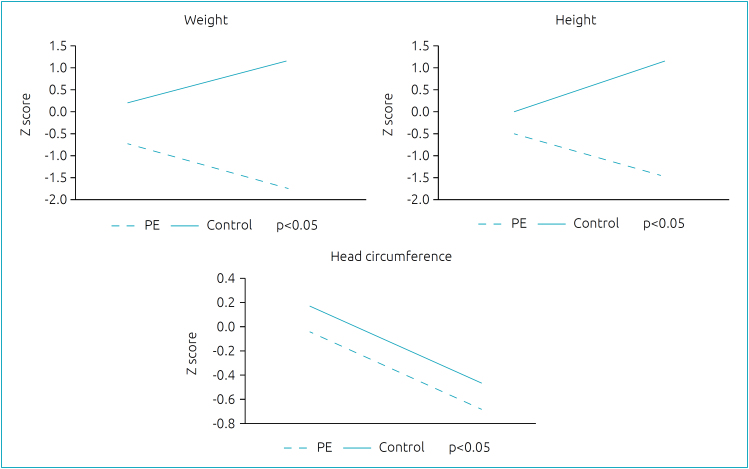
Weight, height, and head circumference Z scores between birth and discharge in preterm of preeclampsia mothers and controls.

The weight and length growth profile of preterm infants, assessed by Z scores at birth and at discharge, was worse in the PE group compared to the control group, but HC growth did not differ (Figure 1).

## DISCUSSION

Despite concerns regarding intestinal hypoperfusion in the infants of PE mothers, in this study, we have shown that early-onset PE was not an independent risk factor for feeding intolerance in preterm infants. Furthermore, we found that gestational age was the most significant predictor of feeding outcome during the neonatal period.

Our patients had very low gestational age; as a result, a delayed introduction of feeds and problems in the advancement of enteral nutrition were expected and occurred in both groups. A concerning finding was the high frequency (>50%) of feeding intolerance in the first week, and in about 40% of the infants, the problems persisted until the end of the first month of life. Feeding intolerance is a great challenge in clinical practice, since it may be associated with NEC which increases morbidity and mortality of preterm infants.^15,16^ However, in this study, despite the high incidence of feeding intolerance, the rate of NEC was low, suggesting that the gastrointestinal symptoms, mainly gastric residual volumes, usually interpreted as feeding intolerance, may be physiological and related to gastrointestinal immaturity and poor gastric motility.^17^ According to our results, we do not recommend routine evaluation of gastric residual volumes.

Clinical instability is common in preterm infants during the first days of life and has an adverse effect on the introduction and advancement of enteral feedings.^18^ In this study, we have shown that enteral feeding was started on day 2, and a high SNAPPE score (>40) was a risk factor for feeding interruption in the first 3 days of life; however, it was a week predictor, increasing only 4% the risk (p=0.05).

A concerning finding in our study was that SGA neonates showed an increased risk of feeding intolerance after the first week of life.^19^ Similarly, a study by Bozzetti et al.^20^ evaluated feeding tolerance of preterm infants appropriate for gestational age as compared to those SGA and found that SGA had a later onset of enteral feeding (5 vs. 3 days), slower feeding advancement (21 vs. 18 days), and spent more time to reach full enteral feeds (27 vs. 21 days). These results are not unexpected in SGA infants and may be ascribed to a chronic prenatal intestinal hypoxic/ischemic injury, leading to impaired gut function after birth and feeding intolerance.

PE is associated with an increased risk of fetal growth restriction.^3^ In this study, we have shown a high percentage of SGA preterm infants in the PE group, which may have contributed to the longer time to achieve full enteral feeding and worse growth during hospitalization in the PE group. The literature is not clear whether the food intolerance in premature infants of mothers with PE is directly related to maternal disease or an indirect effect of this is associated with fetal growth restriction.

In our study, feeding intolerance was associated with being born SGA, but not with PE. Interestingly, NEC was uncommon and not associated with PE, suggesting that nutritional practices are more important than perinatal factors in the nutritional outcome of premature infants. This lack of association must be interpreted with caution due to the small sample size, and further studies are needed to confirm whether there is a direct association of PE with feeding intolerance and NEC. The relationship between perinatal factors and neonatal feeding problems is controversial. A case–control study to investigate maternal risk factors for NEC identified fetal growth restriction and birth weight as risk factors, whereas PE did not show an increased risk of NEC.^21^ In a large cohort of 4,649 preterm infants <32 weeks investigating the epidemiology and risk factors for NEC, the maternal hypertensive disease was found to be protective, reducing the risk of NEC by 39%.^22^

In our study, antenatal MgSO_4_ was associated with a reduced risk for feeding interruption in the first week of life. This result suggests that the effect of PE on the premature infant should be interpreted with caution because it is uncertain whether the neonatal outcome is determined by maternal disease or may be at least partially related to its treatment.^5^ MgSO_4_ decreases vascular tone, with a dose-dependent vasodilator effect on the maternal cerebral and gastrointestinal vascular bed. There is evidence of a beneficial effect of antenatal MgSO_4_ on fetal cerebral circulation, and its use for neuroprotection has been recommended since it reduces the risk of cerebral palsy.^23^ However, there are few reports of its effects on fetal gastrointestinal circulation.

In a case–control study that investigated the effects of antenatal MgSO_4_ on intestinal blood flow and feeding tolerance of 50 preterm infants <34 weeks, the exposure to antenatal MgSO_4_ did not affect intestinal blood flow, and there was no difference regarding time to reach full enteral feeds, first meconium passage, and feeding intolerance.^24^

There is some concern regarding the safety of antenatal MgSO_4_ exposure. An increased risk of spontaneous intestinal perforation was reported in preterm infants weighing <1000g,^24^ but this finding was not confirmed in a large cohort of extremely preterm infants.^25^ Randomized controlled trials and systematic reviews do not support a clear association between antenatal MgSO_4_ and adverse neonatal outcome, suggesting that antenatal MgSO_4_ is safe.^26–30^ Our findings suggesting a beneficial effect of MgSO_4_ are promising; however, further studies are needed to confirm this benefit.

Our study highlights the major influence of gestational age on feeding progression and nutritional outcome. Our results suggest that during the first month of life, feeding tolerance improves as gestational age increases. In both groups, the number of preterm infants without symptoms of intolerance increased from 1–3 days of life to 8–28 days, with statistical significance in PE group (p=0.044). The literature demonstrates the impact of gestational age on feeding outcomes and the importance of low gestation as a risk factor for NEC.^31^ The high proportion of breast milk or human donor milk use during the introduction and progression of feeds may have contributed to the low incidence of NEC in our patients. However, this result should be interpreted with caution since our sample size was not calculated to detect an uncommon event.

The growth trajectory during hospitalization differed between groups, with poor postnatal growth in the PE group. We speculate that several factors could contribute to this outcome, including the higher percentage of SGA, more days of parenteral nutrition, and longer time of achieving full enteral nutrition in the PE group. HC growth did not differ between groups, and HC Z scores decreased from birth to discharge; however, in both groups, the mean scores were in the normal range. Poor postnatal growth is a common problem for VLBW infants, and this issue deserves attention, mainly the head growth that is associated with neurodevelopmental outcomes.^32^

The main limitation of this study is the small sample size and consequently the insufficient power for uncommon outcomes such as enterocolitis and death. Strengths of our study include the prospective design, with strict pairing between the groups and detailed assessment of feeding difficulties over time periods. This design allowed us to identify specific risk factors in each period, showing the dynamic feeding outcome of preterm infants during the first month of life. These findings may help clinicians to better interpret the feeding intolerance of preterm infants. We could not confirm a negative effect of PE on feeding tolerance, and further studies with a large sample are recommended to answer this question.

Preterm infants of mothers with early-onset PE were more likely to be born SGA and had a worse growth trajectory in the NICU. In adjusted analyses, however, low gestational age and SGA were independent predictors of feeding intolerance.
